# High dose antipsychotic polypharmacy and dopamine partial agonists - time to rethink guidelines?

**DOI:** 10.1177/02698811211026456

**Published:** 2021-07-14

**Authors:** Gavin P Reynolds

**Affiliations:** Biomolecular Sciences Research Centre, Sheffield Hallam University, Sheffield, United Kingdom

**Keywords:** Antipsychotic agents, adjunctive treatment, dopamine D2 receptor, antagonists, partial agonists

## Abstract

Guidelines for the treatment of schizophrenia limit the use of antipsychotic agents to clinically-established maximum doses. This acknowledges both the absence of additional efficacy of dopamine D2 receptor antagonists above a receptor occupancy threshold, and the increases in side effects that can occur at higher doses. These limits restrict the dosing of combinations of antipsychotics as they do single agents; drugs sharing the major antipsychotic mechanism of D2 receptor antagonism will act additively in blocking these receptors.

Several newer antipsychotic drugs, including aripiprazole and cariprazine, act as partial agonists at the D2 receptor site and avoid action at several other receptors, effects at which are responsible for some non-dopaminergic adverse effects. This pharmacology imparts different characteristics to the drugs resulting often in a more favourable side effect profile. Their partial agonism, along with high affinities for the D2 receptor, also means that these drugs given adjunctively may in part replace, rather than enhance, the D2 antagonism of other antipsychotic agents. This can result in an improvement in certain side effects without loss of antipsychotic efficacy.

This article makes the case for distinguishing the D2 partial agonists from antagonists in defining maximum doses of combined treatments, which would increase the options available to the prescriber, emphasising that pharmacological mechanisms need to be understood in identifying optimal treatments for psychotic illness.

The dopamine D2 antagonists used as antipsychotic treatments are invaluable for many people with schizophrenia but can be limited by a range of adverse effects. These depend on the pharmacological profile of the antipsychotic drug used, as well as on genetic and other individual features of the patient, and include *inter alia* extrapyramidal symptoms (EPS), elevated prolactin, sedation, postural hypotension, weight gain and QT interval prolongation. The underlying receptor mechanisms relate to antagonism at, respectively but not exclusively, striatal and pituitary D2 receptors, histamine H1, alpha1 adrenergic, H1 and 5-HT2C receptors and hERG ion channels. In general, these side effects are dose-dependent, consideration of which has prompted the introduction of guidelines aimed at avoiding excessively high doses of antipsychotic drug. Furthermore, it has been established that for most dopamine D2 antagonists a D2 receptor blockade of approximately 60%–70% is adequate for an antipsychotic action and that drug doses above those required to achieve this threshold are likely to increase side effects without a concomitant increase in efficacy (Farde et al., 1992; Kapur et al., 2000).

This argument is also true for combinations of D2 antagonist drugs; the UK consensus guidelines indicate that the ‘high dose’ threshold is reached when the sum of the percentage of the maximum recommended dose for each antipsychotic drug exceeds 100% (Royal College of Psychiatrists, 2014). The rationale is clear: Combining two D2 antagonists at half maximum dose should approximate to the effects of one drug at maximum dose, although it is limited by inconsistencies in determining maximum doses, with newer drugs generally having more constrained dose ranges.

The availability of aripiprazole and cariprazine, as well as brexpiprazole in many countries outside the UK, has provided psychiatry with useful alternatives to the various dopamine D2 receptor antagonists that have been the mainstay in the treatment of psychosis for over 60 years. These three drugs are dopamine D2 receptor partial agonists which also have some further pharmacological properties; for aripiprazole this includes 5-HT1A partial agonism and 5-HT2A antagonism, whilst cariprazine has a partial agonist affinity for D3 higher than that for D2 and antagonist action at 5-HT2B (Frankel and Schwartz, 2017). Both drugs have minimal or, at most, weak effects at histamine H1, muscarinic or adrenergic receptors.

These pharmacological profiles have resulted in the availability of antipsychotic pharmacotherapy that is relatively free of some limiting side effects including hyperprolactinaemia and QT prolongation, as well as reductions in sedation (particularly for cariprazine) and in the requirement for antiparkinsonian medication (particularly for aripiprazole) (Huhn et al., 2019).

There is little evidence to support switching between antipsychotic drugs to improve efficacy (Barnes et al., 2020), although recent studies have indicated some differences in which amisulpride, olanzapine and risperidone, as well as clozapine, show small advantages in overall efficacy (Smith et al., 2019). Stronger justification for switching is found in response to side effects which may differ substantially between drugs. Here the D2 partial agonists may demonstrate particular benefits. With these drugs, however, switching from a D2 antagonist may not be immediately necessary; the benefits of aripiprazole (and likely cariprazine too) in reducing drug-induced hyperprolactinaemia, for example, can be seen when the drug is added to current treatment (Zheng et al., 2019). Similarly, adjunctive aripiprazole can ameliorate weight gain and other metabolic problems associated with olanzapine or clozapine treatment (Cooper et al., 2016; Mizuno et al., 2014). Usually there is no deterioration of psychotic symptoms associated with adding a dopamine partial agonist; further symptomatic improvement may even be seen (e.g. Zheng et al., 2019). Such adjunctive prescribing would be off-label but has a strong evidence base. It could, however, also serve as an initial step towards D2 partial agonist monotherapy, prior to a gradual withdrawal of the D2 antagonist drug.

Combination of two antipsychotic agents in this way may exceed the high dose threshold. Such cumulative doses are perceived as high risk and understandably may require explicit justification for their prescription. British Association for Psychopharmacology guidelines indicate that antipsychotic augmentation to address inadequate symptom response should only be considered after other treatment options have been exhausted, including ‘several, adequate, sequential trials of antipsychotic monotherapy’ (Barnes et al., 2020). This both reflects and guides current UK practice where switching is generally preferred to augmentation with another antipsychotic drug, despite the acknowledged paucity of supportive evidence. This often means that drug combinations are avoided with alternative, but perhaps sub-optimal, strategies being employed.

As mentioned above, the pharmacological basis for this high dose risk is clear; additional blockade of D2 receptors above what is necessary for an antipsychotic response will increase the liability for dopaminergic side effects such as EPS and prolactin secretion without increasing efficacy. Furthermore, and depending on the particular drugs being combined, other receptor-mediated side effects are likely to increase, some of which, such as metabolic effects and QT prolongation, may contribute to increased mortality. However, this pharmacological argument does not consistently hold up for the combination of partial agonists with antagonists. In terms of the main antipsychotic mechanism, drug action at the dopamine D2 receptor, the dopamine D2 partial agonists should be differentiated from D2 antagonists. As can be seen in their effects in avoiding, and reversing, D2 antagonist-induced hyperprolactinaemia, adjunctive partial agonists can ameliorate, rather than enhance, unwanted dopaminergic effects of D2 antagonists. Aripiprazole and cariprazine have high (sub-nanomolar) affinities at the D2 receptor greater than most D2 antagonists (Frankel and Schwartz, 2017). Thus they will preferentially bind to the receptor site, resulting in replacing antagonism with partial agonism, thereby providing a level of receptor stimulation which depends on the drug’s intrinsic activity; for aripiprazole this is around 25% of that of dopamine (Cosi et al., 2006).

This provides a strong argument for differentiating the D2 partial agonists from the D2 antagonist drugs and considering them a separate class when it comes to combining drugs for the optimal treatment of people with psychotic illness. Of course, polypharmacy is to be avoided where possible and drugs need to be given at the lowest dose consistent with optimising efficacy and minimising side effects. However, it is clearly wrong to restrict adjunctive use of D2 partial agonists, where they may have clinical value, on the basis of the inappropriate application of a ‘maximum cumulative dose’ concept.

As a postscript, I suggest that the issue highlighted here is related to the common view of ‘antipsychotic agents’ as a single drug class or, at best, one divided into first- and second-generation antipsychotics. The application of neuroscience-based nomenclature, in which drugs are differentiated on the basis of their pharmacological mechanisms (Nutt and Blier, 2016), takes one step towards avoiding this oversimplification and its consequences for psychiatric pharmacotherapy.
