# Supplementary material for: High dose antipsychotic polypharmacy and dopamine partial agonists - time to rethink guidelines?
Source: J Psychopharmacol. 2021 Jul 14;35(9):1030–6. doi: 10.1177/02698811211026456 (PMC8436307; doi:10.1177/02698811211026456)
Supplement: Related texts

# Other: Commentary on ‘High dose antipsychotic polypharmacy and dopamine partial agonists – time to rethink guidelines?’ by Prof Gavin Reynolds

**Published:** 2021-07

Carol Paton. Joint-head. Prescribing Observatory for Mental Health. Honorary Research Fellow. Division of Psychiatry, Imperial College London, Du Cane Road, London W12 ONN, United Kingdom. Carol.Paton@nhs.net

Thomas R. E. Barnes. Professor emeritus of clinical psychiatry. Division of Psychiatry, Imperial College London, London, United Kingdom

Clinical guidelines for the treatment of schizophrenia consistently recommend the use of a single antipsychotic in a standard dose (Barnes et al., 2020; National Institute for Health and Care Excellence, 2014; Galletly et al., 2016); this strategy is underpinned by both a large body of evidence supporting the efficacy of individual antipsychotic medications and by tolerability data that have been systematically, although not always comprehensively, garnered (Pope et al., 2010). These guidelines further recommend switching to an alternative antipsychotic medication if the first antipsychotic is not tolerated or the illness fails to respond, although the paucity of supportive evidence for such switching is acknowledged. Should there be a persistent lack of adequate therapeutic response, timely progression to clozapine treatment should be the next step, in advance of the use of high-dosage antipsychotic medication or combined antipsychotic medication strategies (which commonly result in a high-dose regimen), for which the evidence regarding treatment outcomes and safety is considerably slimmer.

Reynolds argues that, when added to another antipsychotic medication, D_2_ partial agonists reduce metabolic and prolactin-related side effects and potentially improve symptom control: therefore the use of such combinations should be considered earlier when seeking to optimise treatment for schizophrenia. But any additional beneficial effect on symptoms and side-effects with a D_2_ partial agonist/D_2_ antagonist combination is likely to be very modest and the combination may be associated with new side effects, so a favourable balance of risks and benefits cannot be assumed. Most of the clinical literature relating to D_2_ partial agonists focuses on aripiprazole as this antipsychotic medication was first in class. Therefore, aripiprazole is used to expand on these points below.

## Efficacy and tolerability of aripiprazole as monotherapy

While aripiprazole has evident antipsychotic efficacy, it may be slightly less effective overall for treating the symptoms of schizophrenia than the more commonly used D_2_ antagonists (Leucht et al., 2013; Tiihonen et al., 2019), suggesting that D_2_ partial agonism may provide less than optimal control of symptoms in some cases. There are a further group of patients whose illness has shown an insufficient response to non-clozapine antipsychotic medication that is not related to factors such as poor adherence or comorbid substance use. Such treatment-resistant schizophrenia may have a distinct pathophysiology involving a range of neurobiological pathways (Potkin et al., 2020). Therefore, pharmacological strategies that are limited to modulating D_2_ receptor activity, either as monotherapy or in combination, may have minimal if any effect on symptoms and pursuing such strategies could result in an avoidable delay in initiating clozapine treatment, potentially worsening outcomes (Shah et al., 2018; Yoshimura et al., 2017).

When used alone, aripiprazole is undoubtedly relatively well tolerated with respect to metabolic and prolactin-related side effects (Leucht et al., 2013) but it is not free of side effects, with treatment-emergent akathisia, insomnia, headache and nausea reported for more than 1 patient in 10 in clinicial trials (Aripiprazole SmPC). With respect to QTc prolongation, epidemiological studies have linked aripiprazole to weak/moderate tosardogenicity (Polcwiartek et al., 2015). Further, recent evidence points to an association between D_2_ partial agonists and the development of impulse control disorders (Corbeil et al., 2021; Grall-Bronnec et al., 2016). Therefore, as is the case for all antipsychotic medications, the decision to prescribe aripiprazole should take into consideration the potential harms associated with this medication and ongoing clinical review is essential.

## Efficacy and tolerability of aripiprazole in combination with another antipsychotic medication

While to our knowledge there are no randomised controlled trials of aripiprazole augmentation of non-clozapine antipsychotic medication, the real life effectiveness of such combinations has been reported by Tiihonen et al. (2019). In a Swedish, population-based cohort of 62,500 patients with an established diagnosis of schizophrenia, those patients who were prescribed clozapine, olanzapine or a long-acting injectable antipsychotic preparation as monotherapy were less likely to be readmitted to hospital than those prescribed aripiprazole combined with a non-clozapine antipsychotic. Further, olanzapine monotherapy fared slightly better than olanzapine combined with aripiprazole. Thus, these findings failed to suggest any systematic efficacy advantage for the addition of aripiprazole to a non-clozapine antipsychotic medication.

With respect to aripiprazole augmentation of clozapine, a meta-analysis of mostly very small randomised controlled trials found no advantage with respect to psychotic symptoms in clozapine responders who received aripiprazole as a strategy to manage weight gain but a potential modest advantage with respect to improvement in negative symptoms where the illness had not responded to clozapine (Srisurapanont et al., 2015). In the naturalistic study by Tiihonen et al. (2019) described above, the combination of clozapine and aripiprazole was most effective in preventing readmission but, compared with clozapine monotherapy, the effect size was small. When considered together, these studies do not exclude a modest advantage for an individual treatment trial of the combination over clozapine monotherapy for some particular patients, but the evidence so far falls short of supporting routine use.

Aripiprazole binds strongly to D_2_ receptors but the degree of dopamine blockade it produces is limited as it is a partial agonist rather than an antagonist. Through this mechanism, adjunctive aripiprazole has been shown to reverse the hyperprolactinaemia that is associated with some potent D_2_ antagonists, such as risperidone (Yasui-Furukori et al., 2010). However hyperprolactinaemia that is asymptomatic does not always require treatment and where it is symptomatic or the patient is otherwise at greater risk of sequelae, the most logical strategy would be to switch to an antipsychotic medication with a low propensity for elevating plasma prolactin.

With respect to the amelioration of weight gain associated with antipsychotic treatment, published meta-analyses (Choi, 2015; Srisurapanont et al., 2015) have concluded that the addition of aripiprazole to clozapine or olanzapine results in a mean weight change of around -2 kg compared with clozapine or olanzapine monotherapy. This mean change is modest and while it does not exclude a larger effect in some patients, it suggests that for the majority the effect on body weight is unlikely to be clinically relevant. The use of adjunctive aripiprazole to reduce weight gain in patients receiving antipsychotic medications other than clozapine or olanzapine has not been systematically tested. While the BAP guidelines for the management of weight gain, metabolic disturbances and cardiovascular risk associated with psychosis and antipsychotic treatment (Cooper et al, 2016) identified augmentation of clozapine or olanzapine with aripiprazole as a possible intervention for weight gain, it added the caveat that the possible disadvantages of such polypharmacy should be considered versus the degree of benefit obtained. For example, the meta-analysis by Choi (2015) also noted that there were clear signals for treatment-emergent akathisia and nausea, along with one report of sinus tachycardia and two of significant worsening of psychotic symptoms in the aripiprazole augmentation arms.

The meta-analysis by Srisurapanont (2015) reported modest improvement in LDL cholesterol with aripiprazole augmentation but did not find any beneficial effects of the combination with respect to fasting plasma glucose, although it should be noted that the durations of the included studies were short, ranging from 8 to 24 weeks.

## Definition of high-dose antipsychotic medication and recommendations in evidence-based clinical guidelines

A number of approaches have been used to determine whether a combination of antipsychotic medication is considered to be high dose or not (Barnes et al., 2014); the most common are (1) the use of ‘chlorpromazine equivalents’ where the doses of each antipsychotic medication are converted to an equivalent dose of chlorpromazine, based partially on known affinity for D_2_ receptors, and a total equivalent dose of chlorpromazine above 1000 mg is defined as a high dose, and; (2) the more commonly used ‘percentage method’ where the percentages of the maximum licensed dose for each antipsychotic medication co-prescribed are added and a cumulative dose of more than 100% is defined as a high dose. While both methods are essentially arbitrary and have their limitations, they provide a dosage threshold above which the evidence suggests that the risk-benefit balance becomes more uncertain and potentially unfavourable (Leucht et al., 2020) and which should be a prompt that such regimens should be prescribed as individual therapeutic trials, with close monitoring of target symptoms and potential side effects. Guideline recommendations relating to the use of high-dose or combined antipsychotic medication strategies are not based simply on whether a dosage threshold has been exceeded, but consider the available evidence relating to efficacy and safety, to inform the rational prescription of such regimens. Nevertheless, as the evidence base is constantly developing and changing, there is a need to keep all guideline recommendations under review.

## Conclusion

Where evidence-based medication strategies (including clozapine) for schizophrenia have failed or are not tolerated, an individual therapeutic trial of combined antipsychotic medications is a potential treatment option. In this context, a trial of aripiprazole augmentation of another antipsychotic medication may offer worthwhile benefits for some patients in some clinical circumstances. However, given the relatively poor evidence base underpinning the use of combined antipsychotic medications, the advice that other pharmacological interventions with more consistent evidence for a favourable risk-benefit balance should be given priority and that if such a regimen is prescribed there is a responsibility to document the rationale for the chosen combination and carefully monitor the benefits and harms, should support judicious prescribing.

## References

[bibr13-02698811211026456] BarnesTREDrakeRPatonC, et al. (2020) Evidence-based guidelines for the pharmacological treatment of schizophrenia: Updated recommendations from the British Association for Psychopharmacology. J Psychopharmacol34: 3–78.3182977510.1177/0269881119889296

[bibr14-02698811211026456] BarnesTREDyeSFerrierN, et al. (2014) Consensus statement on high-dose antipsychotic medication. Royal College of Psychiatrists. College Report CR190.

[bibr15-02698811211026456] ChoiYJ (2015) Efficacy of adjunctive treatments added to olanzapine or clozapine for weight control in patients with schizophrenia: A systematic review and meta-analysis. Sci World J 2015: 970730.10.1155/2015/970730PMC431026525664341

[bibr16-02698811211026456] CooperSJReynoldsGPBarnesTRE, et al. (2016) BAP guidelines on the management of weight gain, metabolic disturbances and cardiovascular risk associated with psychosis and antipsychotic drug treatment. J Psychopharmacol30: 717–748.2714759210.1177/0269881116645254

[bibr17-02698811211026456] CorbeilOCorbeilSDorvalM, et al. (2021) Problem gambling associated with aripiprazole: A nested case-control study in a first-episode psychosis program. CNS Drugs35: 461–468.3371329810.1007/s40263-021-00801-4

[bibr18-02698811211026456] GalletlyCCastleDDarkF, et al. (2016) Royal Australian and New Zealand College of Psychiatrists clinical practice guidelines for the management of schizophrenia and related disorders. Aust N Z J Psychiatry50: 410–472.2710668110.1177/0004867416641195

[bibr19-02698811211026456] Grall-BronnecMSauvagetAPerrouinF, et al. (2016) Pathological gambling associated with aripiprazole or dopamine replacement therapy. Do patients share the same features? A review. J Clin Psychopharmacol36; 63–70.2665826310.1097/JCP.0000000000000444PMC4700874

[bibr20-02698811211026456] LeuchtSCiprianiASpinelliL, et al. (2013) Comparative efficacy and tolerability of 15 antipsychotic drugs in schizophrenia: A multiple-treatments meta analysis. Lancet382: 951–962.2381001910.1016/S0140-6736(13)60733-3

[bibr21-02698811211026456] LeuchtSCrippaASiafisS, et al. (2020) Dose-response meta-analysis of antipsychotic drugs for acute schizophrenia. Am J Psychiatry177: 342–353.3183887310.1176/appi.ajp.2019.19010034

[bibr22-02698811211026456] National Institute for Health and Care Excellence (2014) Psychosis and schizophrenia in adults: Prevention and management. Clinical Guideline 178. 2014. Available at: https://www.nice.org.uk/guidance/cg17832207892

[bibr23-02698811211026456] PolcwiartekCSneiderBGraffC, et al. (2015) The cardiac safety of aripiprazole in patients at high risk for torsade: A systematic review with a meta-analytic approach. Psychopharmacology232: 3297–3308.2623149710.1007/s00213-015-4024-9

[bibr24-02698811211026456] PopeAAdamsCPatonC, et al. (2010) Assessment of adverse effects in clinical studies of antipsychotic medication: Survey of methods used. Br J Psychiatry197: 67–72.2059243810.1192/bjp.bp.109.070961

[bibr25-02698811211026456] PotkinSGKaneJMCorrellCU, et al. (2020) The neurobiology of treatment-resistant schizophrenia: Paths to antipsychotic resistance and a roadmap for future research. NPJ Schizophr6: 1.3191162410.1038/s41537-019-0090-zPMC6946650

[bibr26-02698811211026456] ShahPIwataYPlitmanE, et al. (2018) The impact of delay in clozapine initiation on treatment outcomes in patients with treatment-resistant schizophrenia: A systematic review. Psychiatry Res268: 114–122.3001510910.1016/j.psychres.2018.06.070

[bibr27-02698811211026456] SrisurapanontMSuttajitSManeetonN, et al. (2015) Efficacy and safety of aripiprazole augmentation of clozapine in schizophrenia: A systematic review and meta-analysis or randomized-controlled trials. J Psychiatr Res62: 38–47.2561917610.1016/j.jpsychires.2015.01.004

[bibr28-02698811211026456] Summary of Product Characteristics. Aripiprazole. Electronic Medicines Compendium. Available at: https://www.medicines.org.uk/emc/product/7969/smpc#UNDESIRABLE_EFFECTS

[bibr29-02698811211026456] TiihonenJTaipaleHMehtalaJ, et al. (2019) Association of antipsychotic polipharmacy vs monotherapy with psychiatric rehospitalization among adults with schizophrenia. JAMA Psychiatry76: 499–507.3078560810.1001/jamapsychiatry.2018.4320PMC6495354

[bibr30-02698811211026456] UcokAUgurCKarabulutS, et al. (2015) Delayed initiation of clozapine may be related to poor response to treatment-resistant schizophrenia. Int Clin Psychopharmacol30: 290–295.2616387510.1097/YIC.0000000000000086

[bibr31-02698811211026456] Yasui-FurukoriNFurukoriHSugawaraN, et al. (2010) Dose dependent effects of adjunctive treatment with aripiprazole on hyperprolactinaemia induced by risperidone in female patients with schizophrenia. J Clin Psychopharmacol30: 596–599.2081433310.1097/JCP.0b013e3181ee832d

[bibr32-02698811211026456] YoshimuraBYadaYSoR, et al. (2017) The critical treatment window of clozapine in treatment-resistant schizophrenia: Secondary analysis of an observational study. Psychiatry Res250: 65–70.2814206810.1016/j.psychres.2017.01.064

# Other: Response to Paton and Barnes

**Published:** 2021-07

Arguably the biggest concern in schizophrenia research is that of people with schizophrenia whose symptoms do not respond to standard antipsychotic agents (James Lind Alliance, 2011). Until more effective drugs are introduced which address this problem, it is important to provide a therapeutic environment that offers clinicians and their patients the most flexibility in treatment choice without restrictions that are not firmly grounded in evidence. To my mind, this includes the opportunity to provide a relatively safe treatment with a D2 partial agonist additional to a D2 antagonist, while recognising of course that, as with all pharmacotherapy, there is a balance to be struck between risk and benefit.

Paton and Barnes have prepared an extensive response to my commentary, starting by summarising the guidelines for the treatment of schizophrenia. These recommend monotherapy and switching to an alternative antipsychotic agent if this is not tolerated or if the illness fails to respond. Only the first of these reasons to switch has a strong evidence base. They also discuss at length the evidence for the efficacy and tolerability, or otherwise, of aripiprazole treatment for schizophrenia. They repeatedly describe benefits of adjunctive aripiprazole as modest; however, less than modest supporting evidence is no barrier to inclusion in guidelines, as we see with the recommendation to switch drugs for lack of response. We can recognise this as a pragmatic recommendation – ‘what else can be done?’ - in the hope that some individual factor may result in improvement, but evidence suggests it remains unlikely to be effective.

There is much opportunity for further debate around the effects of aripiprazole that Paton and Barnes discuss so comprehensively. One point they make is, however, misleading. They reiterate a comment in the abstract of Polcwiartek et al. (2015) that epidemiological studies have linked aripiprazole to weak/moderate torsadogenicity. Yet the authors also state that a ‘lack of adequate data . . . confounds the interpretation of any epidemiological findings’. More importantly, the main finding of this review and meta-analysis of aripiprazole’s cardiac safety in patients at high risk for torsade is that QTc prolongation risk is lower compared with both placebo and active controls. In a more recent population-based Danish study (Polcwiartek et al., 2020), the same research group report that, while antipsychotic prescribing in people with schizophrenia was associated with a greater frequency of abnormal ECGs compared to non-psychiatric control subjects, this was not true for aripiprazole which was additionally associated with a decreased risk of QTc prolongation. It would be reasonable to conclude that aripiprazole is relatively safe in terms of this measure of cardiac toxicity.

One final point: while I have not specifically considered the adjunctive use of partial D2 agonists with clozapine, Paton and Barnes have helpfully drawn attention to the study of Tiihonen et al. (2019). The key finding from this nationwide Finnish study is that aripiprazole and clozapine polypharmacy is significantly more effective in reducing rehospitalization than clozapine or any antipsychotic monotherapy alone. While the effect size may be small, we are reminded by the authors that this may be an underestimate as adjunctive treatment is generally in response to a worsening of symptoms with monotherapy. Tiihonen et al. (2019) conclude that a rational polypharmacy with two particular drugs of differing receptor profiles appears feasible, and that treatment guidelines which discourage all antipsychotic polypharmacy should be modified accordingly. While this has not been the main point of my commentary, such evidence-based polypharmacy would be facilitated by the removal of a ‘high dose’ restriction on adjunctive D2 partial agonists that has no pharmacological rationale.

## References

[bibr33-02698811211026456] James Lind Alliance (2011) Schizophrenia priority setting partnership. Available at: www.jla.nihr.ac.uk/priority-setting-partnerships/schizophrenia/top-10-priorities/ (accessed 26 April 2021).

[bibr34-02698811211026456] PolcwiartekCSneiderBGraffC, et al. (2015) The cardiac safety of aripiprazole in patients at high risk for torsade: A systematic review with a meta-analytic approach. Psychopharmacology232: 3297–3308.2623149710.1007/s00213-015-4024-9

[bibr35-02698811211026456] PolcwiartekCKragholmKHansenSM, et al. (2020) Electrocardiogram characteristics and their association with psychotropic drugs among patients with schizophrenia. Schizophr Bull46: 354–362.3121959610.1093/schbul/sbz064PMC7442389

[bibr36-02698811211026456] TiihonenJTaipaleHMehtalaJ, et al. (2019) Association of antipsychotic polipharmacy vs monotherapy with psychiatric rehospitalization among adults with schizophrenia. JAMA Psychiatry76: 499–507.3078560810.1001/jamapsychiatry.2018.4320PMC6495354
